# Psychometric Properties and Refinement of the ATT-IPV Scale to Measure Attitudes about Intimate Partner Violence among Married Adolescents and Their Husbands in Niger

**DOI:** 10.3390/ijerph20146385

**Published:** 2023-07-18

**Authors:** Ruvani W. Fonseka, Sneha Challa, Jay G. Silverman

**Affiliations:** 1 Center on Gender Equity and Health (GEH), University of California San Diego School of Medicine, 9500 Gilman Drive, La Jolla, CA 92093, USA; 2 School of Social Work, San José State University, 1 Washington Square, San José, CA 95192, USA; 3 School of Nursing, University of California San Francisco, 3333 California Street, San Francisco, CA 94118, USA

**Keywords:** violence against women and girls, West Africa, ATT-IPV scale, attitudes, gender-based violence, Niger, married adolescents, differential item functioning

## Abstract

(1) Background: This study sought to assess the appropriateness of a five-item scale to measure attitudes towards IPV (ATT-IPV) among married adolescent girls and their husbands in Niger, a population in which this scale has not yet been tested. (2) Methods: Using data collected from 1100 pairs of married adolescents, aged 13–19 years old, and their husbands across 48 villages in rural Niger, we performed classical test theory reliability and exploratory factor analysis, followed by item response theory (IRT) analyses and testing differential item functioning (DIF) by gender. (3) Results: The ATT-IPV scale was found to be internally consistent (alpha = 0.8) and unidimensional in this population, with all items loading onto one factor. We found differential item functioning of the following item: “In your opinion, is a husband justified in hitting or beating his wife in the following situations: If she burns his food?” by gender, suggesting that in order to have a scale that performs similarly in men and women, that item should be removed. (4) Conclusions: The ATT-IPV scale is useful as a measure of attitudes towards IPV among married adolescents and their husbands in Niger. However, it may need to be updated to reflect additional forms of violence and to eliminate gender-differential responses in order to be a more effective measure.

## 1. Introduction

Intimate partner violence (IPV) against women and girls is a pressing public health issue that affects almost 1 in 3 female adolescents (age 15–19) and young women (age 20–24) globally [1]. IPV experience is linked to numerous negative health outcomes, including HIV, unintended pregnancy, depression, and death. Cross-sectional studies in multiple settings have shown women’s experiences of IPV to be associated with them holding attitudes accepting IPV [2,3]. The level of acceptance of IPV within a population may indicate the likelihood of IPV experience in the same population.

Niger, a country in West Africa, has the highest rate of child marriage in the world, with 76% of girls marrying by the age of 18 [4,5]. The acceptance of IPV among married adolescent girls and their husbands might be an important predictor of IPV experience and other gender equity measures, but it has not been measured before in this population. The power differential that exists between husbands and wives, particularly in the context of child marriage, can lead to higher risks of IPV [6,7,8,9,10,11,12]. Differences in the acceptance of IPV may be one way to measure the power dynamic between husbands and wives in this context.

In the field of research on the acceptance of IPV, one set of questions, the ATT-IPV scale, is used frequently as part of the Demographic and Health Surveys administered in many low- and middle-income settings around the world [13,14]. A series of scenarios that survey respondents agree to as acceptable justification for a husband to beat his wife, the ATT-IPV scale has only been psychometrically tested once, among married adults in Vietnam [15], where differential item functioning (DIF) was found between women and men, meaning that certain items in the scale performed differently across gender. This suggests that further studies should be conducted that assess the gender variance of the ATT-IPV scale in other mixed gender populations. The ATT-IPV scale has not yet been psychometrically tested within a West African or married adolescent population.

This study’s aim was to test the psychometric properties of the ATT-IPV scale among married adolescent girls and their husbands in Niger, comparing the scale’s functioning among men and women. The study utilized the secondary data analysis of baseline data collected as part of the Reaching Married Adolescents study in Niger [16]. We hypothesized that the scale would have only one dimension or factor, and that there would be differential item functioning in at least one item across men and women in this sample.

## 2. Materials and Methods

### 2.1. Data Collection

#### 2.1.1. Setting 

Data were collected across 48 villages clustered within the Dosso, Doutchi, and Loga districts in the Dosso region of Niger as part of the baseline data collection for a cluster randomized control trial evaluating family planning intervention [16,17]. Villages were randomly selected based on the following inclusion criteria: (1) having at least 1000 permanent inhabitants; (2) primarily Hausa- or Zarma-speaking; (3) located in Dosso, Doutchi, or Loga districts; and (4) no other NGO intervening specifically around family planning or female empowerment with married adolescent wives or their husbands. Because the baseline data used in this analysis were collected prior to the intervention’s implementation, both intervention and control villages from the study were included.

#### 2.1.2. Participants 

Married female adolescents, aged 13–19 years old (n = 1100), and their husbands (n = 1100) were randomly selected from a list of all eligible married female adolescents provided by each village chief. Eligibility criteria for adolescent wives’ inclusion in the study included: (1) aged 13–19 years old; (2) married; (3) fluent in Hausa or Zarma; (4) residing in the village where recruitment took place with no plans to move away in the next 18 months or plans to travel for more than 6 months during that period; (5) not currently sterilized; and (6) providing informed consent to participate in the study [17].

#### 2.1.3. Survey Administration 

Surveys were conducted by trained research assistants from the Dosso region who could fluently read and speak French and fluently speak Hausa and/or Zarma. Research assistants visited randomly selected households and conducted a Household Recruitment Screener to confirm eligibility. If the household was found not to include an eligible wife and husband, a randomly selected replacement was recruited in their place. Up to three visits were made to each of the selected participants, and if they could not be reached after three attempts, no additional efforts were made [17].

Surveys were administered in a private location (out of earshot of another person, in a place the participant indicated as private) in the village. The research assistant conducting the survey was gender-matched with the participant. Surveys were conducted in either Hausa (31%) or Zarma (69%) language, depending on participant’s language preference. The survey took approximately 40–60 min to complete and was administered using pre-programmed tablets. The encrypted, de-identified data were uploaded via secure internet connection on a weekly basis [17].

#### 2.1.4. Measures 

Survey items for wives and husbands were close-ended questions constructed to reflect the experiences, meanings, and language of the target population, based on formative research findings, prior work of the project team, and existing reliable and validated instruments for men and women in low-resource settings, including the Demographic and Health Survey (DHS) [14,18]. The surveys were developed in English, translated into French, back-translated in English for a content reliability check, programmed in French, and verbally administered in Hausa or Zarma. Due to it being uncommon in the region for bilingual French/Hausa and French/Zarma-speaking research assistants to be able to read Hausa and Zarma, this translation protocol has been commonly utilized in Niger.

#### 2.1.5. The ATT-IPV Scale 

In this study, the ATT-IPV scale was composed of 5 questions common to most DHS evaluations around the world and developed specifically to measure the justification of wife-beating [14]. The 5 questions are listed below.

All were preceded by the phrase “In your opinion, is a husband justified in hitting or beating his wife in the following situations”:

1. If she goes out without telling him?
2. If she uses a family planning method without his permission?
3. If she argues with him?
4. If she refuses to have sex with him?
5. If she burns his food?

All questions gave participants the following answer options: “Agree” or “Yes”, “Disagree” or “No”, “Decline to Answer”, or “Don’t Know”. For the purposes of this analysis “Don’t Know” and “Decline to Answer” were treated as missing responses. Responses of “Agree” or “yes” were coded as 1 and responses of “Disagree” or “no” were coded as 0.

For concurrent validity, additional measures were collected. The first was a 7-question version of the Gender-Equitable Men (GEM) Scale, which has had a Cronbach’s alpha of 0.81 in previous studies [19]. All questions gave participants the following answer options: “Agree”, “Disagree”, “Decline to Answer”, or “Don’t Know.” “Don’t Know” and “Decline to Answer” were treated as missing responses. Responses of “Agree” were coded as 1 and responses of “Disagree” were coded as 0. The seven GEM questions are listed below:

1. A woman’s most important role is to take care of the home and cook for the family.
2. A man should have the final word about decisions in the home.
3. People in my village think that there are times when a woman deserves to be beaten.
4. It is shameful when men engage in caring for children or other domestic work.
5. Giving baths to children, changing children’s clothes, and feeding children are the mother’s responsibility.
6. A woman should never question her husband’s decisions even if she disagrees with them.
7. It is natural and right that men have more power than women in the family.

The second concurrent validity item to be tested was the lifetime experience of physical intimate partner violence. This measure was expected to be correlated with acceptance of IPV, based on previous studies that found associations between these two constructs [2]. Physical IPV was measured as a binary variable, with yes = 1 and no = 0. The physical IPV variable was counted as “yes” if the adolescent wife answered “yes” to any of the following questions. The series of questions was preceded by the phrase “Has your husband ever done any of the following things to you”:

1. Push you, shake you, or throw something at you?
2. Slap you?
3. Twist your arm or pull your hair?
4. Hit you with his fist or with something that could hurt you?
5. Kick you, drag you, or beat you up?
6. Try to choke you or burn you?

### 2.2. Analysis

The male and female data (n = 2200) were randomly split into two halves, a training set, on which most of the classical test theory reliability and exploratory factor analysis was conducted, and a test set, which was used for item response theory (IRT) and testing differential item functioning (DIF) by gender.

#### 2.2.1. Reliability and Dimensionality 

The reliability of the 5 item ATT-IPV scale (Table 1) was tested first using a classical test theory approach, by calculating the Cronbach’s alpha of the scale, as well as Revelle’s omega. An exploratory factor analysis was conducted to assess the dimensionality of the scale. We tested the fit of the 5-item scale within a 1, 2, and bi-factor model. With the second split half or test set, we applied item response theory (IRT) to compare the included items in the test population.

#### 2.2.2. Differential Item Functioning (DIF) 

As our next step, we performed a differential item functioning (DIF) analysis by gender, aiming to follow the methods used by a previous study of the ATT-IPV scale in Vietnam [15]. In the DIF analysis, we performed exploratory factor analysis of the 5-item ATT-IPV scale in two single-gender groups to assess its unidimensionality across men and women. Next, we used the ltm package in R to compare the item information and total test information curves across men and women. We identified any gender-variant items and removed them, testing the adapted scale once more for differential functioning by gender.

#### 2.2.3. Validity 

Face validity was assessed during survey development, when a team of experts agreed on the items to include to measure the acceptance of IPV. It was also implemented in developing the original IPV justification questions included in the DHS [14]. We tested concurrent validity by assessing the level of correlation between the ATT-IPV scale and physical IPV (the type of IPV that the ATT-IPV measures justification of), as well as a GEM scale measuring gender equity. We hypothesized that physical IPV would be more correlated with ATT-IPV than GEM, as this association has been idenitified in previous studies [2]. In addition to testing these correlations in the whole group, we separated the test set by gender and tested these correlations to see if there were any gender differences in concurrent validity.

## 3. Results

### 3.1. Reliability and Dimensionality

The ATT-IPV 5-item scale had a Cronbach’s alpha of 0.8, and the Revelle’s omega alpha was also 0.8. This equivalence between the two alphas suggests that the ATT-IPV scale is likely unidimensional in this population, as the Revelle’s omega alpha is generally lower than the Cronbach’s alpha in multidimensional scales. We next used exploratory factor analysis with the training set of the data to assess the dimensionality, testing one-, two-, and bi-factor models. The results of the various models are presented in Table 2 and Table 3.

**Figure 1:**
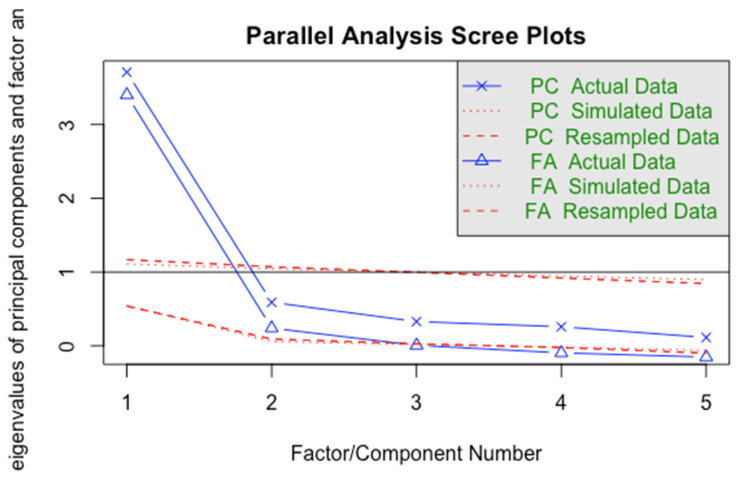
Parallel analysis scree plot.

From the exploratory factor analysis, it was concluded that a one-factor model was most fitting, with all items having factor loading above 0.7 on a shared factor. Additional evidence that supported this was the fact that, in the forced two-factor model, the two factors had a high 0.78 correlation, and in the hierarchical model, the factor eigenvalues were far below 1 (0.34 and 0.27), with the majority of each item’s variance loading on the shared factor. We also tested the factor structure using a Velicer minimum average partial (MAP) analysis and parallel analysis scree plot—both also supported the one-factor model. The Velicer MAP achieved a minimum of 0.09 with one factor, while the parallel analysis scree plot (Figure 1) suggested that a one-factor model was the only model with factor eigenvalues above one.

### 3.2. Item Response Theory 

**Figure 2:**
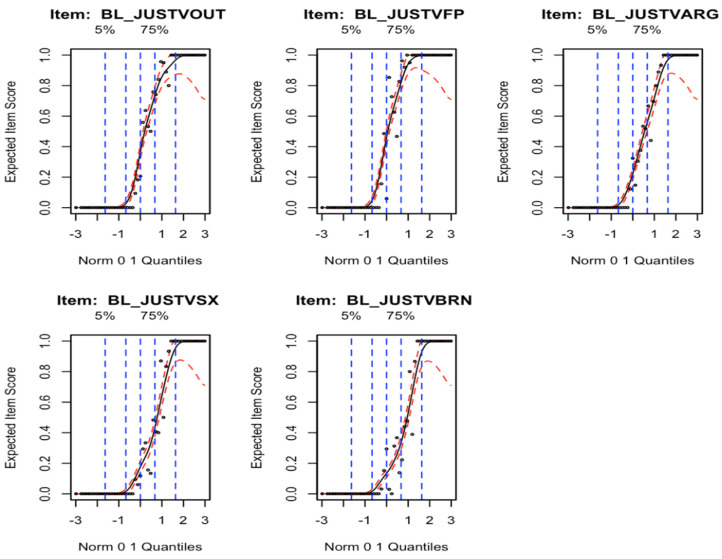
Item Response Curves.

We next applied item response theory (IRT) by comparing the nonparametric expected item score (EIS) curves for each included item (Figure 2) in the test set. Each item had a steep slope, and the five items captured variability above the mean well. However, every EIS curve flattened to zero within one standard deviation below the mean, meaning that none of these items capture variability in those with a low acceptance of IPV in the sample.

### 3.3. Differential Item Functioning (DIF) 

Next, we used the DIF function within mirt to create an “anchor” model that idenitifies the least invariant items and then builds up the model using those items as a starting point. The anchor items with *p*-values greater than 0.05 were BL_JUSTVOUT (*p* = 0.47), BL_JUSTVARG (*p* = 0.34), and BL_JUSTVSX (*p* = 0.79). Setting these three items as the anchor items, we ran a DIF function to assess the DIF of the other two items, BL_JUSTVFP and BL_JUSTVBRN. A table depicting the findings is included (Table 4).

From the model, BL_JUSTVBRN or “In your opinion, is a husband justified in hitting or beating his wife in the following situations: If she burns his food?” had differential item functioning between men and women and should be removed from the scale in order to have a scale that is gender invariant in this population.

### 3.4. Validity 

Face validity for the ATT-IPV scale was assessed during survey development, when a team of experts agreed on including the ATT-IPV scale to measure the acceptance of IPV. It was also implemented in developing the original IPV justification questions included in the DHS (DHS, 2020).

We tested concurrent validity by assessing the level of correlation between the ATT-IPV scale and physical IPV, as well as a scale measuring the gender-equitable man (GEM) scale. In the total group (men and women), the correlation between ATT-IPV and physical IPV was 0.07 (*p* = 0.08), and the correlation between ATT-IPV and the GEM scale (Cronbach’s alpha = 0.46 in this sample) was 0.18 (*p* < 0.01). However, when we separated the group by gender, the results were very different among men and women. Among women, the correlation between ATT-IPV and physical IPV experience was 0.10 (*p* = 0.04), and the correlation between ATT-IPV and GEM was 0.09 (*p* < 0.01), which was the hypothesized outcome. However, with men, the correlation between ATT-IPV and physical IPV perpetration (measured by a wife’s report of experiencing IPV) was 0.02 (*p* = 0.70), and the correlation between ATT-IPV and GEM was 0.27 (*p* < 0.01). It seemed that, in men, ATT-IPV was more correlated with other gender-inequitable attitudes than with perpetrating violence, while in women, ATT-IPV was similarly linked to having experienced violence and holding other gender-inequitable attitudes.

## 4. Discussion 

We found that the original ATT-IPV justification of wife-beating scale was an internally consistent (alpha = 0.8) and unidimensional measure of acceptance of IPV within a population of married adolescents and their husbands in Niger. However, we also found differential item functioning within the ATT-IPV scale for men and women in this sample. In order to have a scale that is less gender-invariant, one item (“In your opinion, is a husband justified in hitting or beating his wife in the following situations: If she burns his food?”) needs to be removed. This item might be interpreted differently by men and women, leading to a difference in response across the same values of the latent trait. One possible explanation for this might be different interpretations of the act of “burning” food by husbands and wives—this item was the only item in which fewer wives justified beating than husbands, and perhaps it is because they interpreted the item as meaning to have accidentally burnt food due to a cooking error, while husbands might have interpreted it as having intentionally burnt his food out of anger.

Our findings relate to the literature on this topic, particularly in differences between men and women around the acceptance of violence. In our sample, women generally endorsed IPV at higher rates than men, except for the one gender-variant item about burning food. This propensity for women to endorse violence at higher rates than men was also found in a study of the ATT-IPV scale in Vietnam, with no exceptions by item [15]. Other studies of this sample have also found men to express more gender-equitable attitudes than their adolescent wives [20], suggesting that, in gender-inequitable contexts such as child marriage, it may be likely that women express more gender-inequitable attitudes, including supporting IPV compared to their husbands. More research on gender differences in the ATT-IPV scale should be conducted in other contexts where child marriage is prevalent.

There were some limitations to this research, namely language and translation issues. The scale was initially conceived in English, then translated to French and finally orally translated in the field to Hausa and Zarma. This might have affected the interpretation of each item, and could have contributed to variations in item performance based on the language of administration or based on individual translations. Back-translation to French of the oral translations could ensure consistency of translation in future research conducted in this way. Future studies should include more ATT-IPV items from the available item pool for cross-validation. The Cronbach alpha of the GEM scale in these samples and the correlations between ATT-IPV and GEM and ATT-IPV and physical IPV were all low, suggesting that the GEM scale might not perform as designed in this population, and that both GEM and physical IPV seem to measure constructs in this population that are less related to ATT-IPV than initially predicted. Finally, testing DIF across genders in this sample might not be meaningful if IPV acceptance is a different latent variable across genders. This is possible since one group (women) generally experiences IPV as an expected victim, and the other group (men) relates to IPV as the expected perpetrator, which is clear from the current wording of the ATT-IPV scale items and was supported by the different correlations between ATT-IPV, GEM, and physical IPV compared across genders.

## 5. Conclusions

This study suggests multiple future directions for research on attitudes towards IPV, particularly among married adolescents in West Africa. First, the gender differences between men and women relating to the scale’s items and the latent construct warrant further investigation. Second, there is a need for comparisons of different items from the full-potential ATT-IPV item set, rather than just the five items that were included in this large survey. These questions have been in use for decades, and with new forms of violence, including cyber sexual harassment, it is important for the ATT-IPV scale to be updated to match the reality of IPV today, recognizing its myriad physical and non-physical forms.

## Figures and Tables

**Table 1:** Variable names, associated scale item, and proportion agreeing ^1^.

Variable Name	Associated Scale Item	Women (n = 704)	Men (n = 704)
BL_JUSTVOUT	If she goes out without telling him?	0.51	0.32
BL_JUSTVFP	If she uses a family planning method without his permission?	0.59	0.33
BL_JUSTVARG	If she argues with him?	0.38	0.28
BL_JUSTVSX	If she refuses to have sex with him?	0.37	0.23
BL_JUSTVBRN	If she burns his food?	0.23	0.28

^1^ These questions in the survey were preceded by the phrase “In your opinion, is a husband justified in hitting or beating his wife in the following situations”.

**Table 2:** One- and two-factor model loadings (standardized loadings (pattern matrix) based upon correlation matrix).

Item	PA1 (Loading on Factor 1)	PA2 (Loading on Factor 2)	h2	u2	com
*One-factor model*					
BL_JUSTVOUT	0.84	-	0.71	0.29	1
BL_JUSTVFP	0.91	-	0.82	0.18	1
BL_JUSTVARG	0.79	-	0.62	0.38	1
BL_JUSTVSX	0.81	-	0.65	0.35	1
BL_JUSTVBRN	0.72	-	0.53	0.48	1
SS loadings	3.32	-	-	-	-
Proportion of variance	0.66	-	-	-	-
*Two-factor model*					
BL_JUSTVOUT	0.90	−0.01	0.80	0.196	1
BL_JUSTVFP	0.90	0.06	0.90	0.099	1
BL_JUSTVARG	0.05	0.81	0.72	0.279	1
BL_JUSTVSX	0.43	0.41	0.63	0.369	2
BL_JUSTVBRN	0.01	0.78	0.62	0.376	1
SS loadings	2.03	1.65	-	-	-
Proportion of variance	0.41	0.33	-	-	-
Cumulative Var	0.41	0.74	-	-	-
Proportion Explained	0.55	0.45	-	-	-
Cumulative Proportion	0.55	1.00	-	-	-
Factor correlation	0.78	-	-	-	-

**Table 3:** Bi-factor model (Schmid Leiman Factor loadings greater than 0.2).

Variable	g	F1	F2	h2	u2	p2
BL_JUSTVOUT	0.67	0.40	-	0.61	0.39	0.74
BL_JUSTVFP	0.72	0.41	-	0.68	0.32	0.76
BL_JUSTVARG	0.61	-	0.35	0.50	0.50	0.76
BL_JUSTVSX	0.59	-	0.20	0.40	0.60	0.85
BL_JUSTVBRN	0.53	-	0.33	0.40	0.60	0.72
eigenvalue	1.96	0.34	0.27	-	-	-

**Table 4:** Differential item functioning output for BL_JUSTVFP and BLJUSTVBRN.

Variable	Gender	AIC	AICc	SABIC	HQ	BIC	logLik	X^2^	df	*p*
BL_JUSTVFP	Female	3547.5	3548.2	3568.2	3573.9	3615.9	−1758.8	-	-	-
BL_JUSTVFP	Male	3549.3	3550.1	3571.5	3577.5	3622.3	−1758.7	4.95	1	0.69
BL_JUSTVBRN	Female	3552.3	3553.0	3573.0	3578.7	3620.6	−1761.1	-	-	-
BL_JUSTVBRN	Male	3549.3	3550.1	3571.5	3577.5	3622.3	−1758.7	4.95	1	0.03

## Data Availability

Due to the sensitivity of the data used for this analysis, additional information, including deidentified participant data and a data dictionary, can be made available upon reasonable request. Please contact the UC San Diego Center on Gender Equity and Health (GEH@ucsd.edu) for any data requests.

## References

[1] Decker M.R., Latimore A.D., Yasutake S., Haviland M., Ahmed S., Blum R.W., Sonenstein F., Astone N.M. (2015). Gender-Based Violence against Adolescent and Young Adult Women in Low- and Middle-Income Countries. J. Adolesc. Health.

[2] Lawoko S. (2008). Predictors of Attitudes toward Intimate Partner Violence. J. Interpers. Violence.

[3] Reese B.M., Chen M.S., Nekkanti M., Mulawa M.I. (2021). Prevalence and Risk Factors of Women’s Past-Year Physical IPV Perpetration and Victimization in Tanzania. J. Interpers. Violence.

[4] Institut National de la Statistique (INS) and ICF International Enquête (2013). Enquête Démographique et de Santé et à Indicateurs Multiples du Niger 2012.

[5] World Health Organization (2021). WHO African Region Fact Sheet Violence against Women Prevalence Estimates 2018.

[6] Speizer I.S., Pearson E. (2011). Association between Early Marriage and Intimate Partner Violence in India: A Focus on Youth from Bihar and Rajasthan. J. Interpers. Violence.

[7] Raj A. (2010). When the mother is a child: The impact of child marriage on the health and human rights of girls. Arch. Dis. Child..

[8] Raj A., Jackson E., Dunham S. (2017). Girl Child Marriage: A Persistent Global Women’s Health and Human Rights Violation. Global Perspectives on Women’s Sexual and Reproductive Health Across the Lifecourse.

[9] Nour N. (2006). Health consequences of child marriage in Africa. Emerg. Infect. Dis..

[10] Tenkorang E.Y. (2019). Explaining the links between child marriage and intimate partner violence: Evidence from Ghana. Child Abus. Negl..

[11] Kidman R. (2017). Child marriage and intimate partner violence: A comparative study of 34 countries. Int. J. Epidemiol..

[12] Male C., Wodon Q. (2018). Girls’ Education and Child Marriage in West and Central Africa: Trends, Impacts, Costs, and Solutions. Forum Soc. Econ..

[13] Hindin M.J., Kishor S., Ansara D.L. (2008). Intimate Partner Violence among Couples in 10 DHS Countries: Predictors and Health Outcomes.

[14] DHS (2020). Demographic Health Survey Questionnaires and Modules: Domestic Violence Module.

[15] Yount K.M., VanderEnde K., Zureick-Brown S., Anh H.T., Schuler S.R., Minh T.H. (2014). Measuring Attitudes About Intimate Partner Violence Against Women: The ATT-IPV Scale. Demography.

[16] Challa S., Delong S.M., Carter N., Johns N., Shakya H., Boyce S.C., Vera-Monroy R., Aliou S., Ibrahima F.A., Brooks M.I. (2019). Protocol for cluster randomized evaluation of reaching married adolescents-a gender-synchronized intervention to increase modern contraceptive use among married adolescent girls and young women and their husbands in Niger. Reprod. Health.

[17] Shakya H., Weeks J.R., Fleming P.J., McDougal L., Scobel A., Cislaghi B., Boyce S., Raj A., Silverman J. The association between individual and village level demographic characteristics and age at first marriage among married adolescents in rural Niger: A spatial analysis. Proceedings of the Population Association of America (PAA) Annual Meeting.

[18] Barker G., Contreras J.M., Heilman B., Singh A.K., Verma R.K., Nascimento M. (2015). Evolving Men: Initial Results from the International Men and Gender Equality Survey (IMAGES), 2011.

[19] Pulerwitz J., Barker G. (2008). Measuring attitudes toward gender norms among young men in Brazil: Development and psychometric evaluation of the GEM scale. Men Masc..

[20] Fonseka R.W., DeLong S.M., Shakya H.B., Challa S., Brooks M.I., Silverman J.G. (2022). Associations between spousal gender equity and recent unintended pregnancy among married adolescent girls and their husbands in rural Niger. Afr. J. Reprod. Health.

